# Harnessing male germline epigenomics for the genetic improvement in cattle

**DOI:** 10.1186/s40104-023-00874-9

**Published:** 2023-06-06

**Authors:** Xiao Wang, Wenlong Li, Xia Feng, Jianbin Li, George E. Liu, Lingzhao Fang, Ying Yu

**Affiliations:** 1 Laboratory of Animal Genetics and Breeding, Ministry of Agriculture and Rural Affairs of China, National Engineering Laboratory of Animal Breeding, College of Animal Science and Technology, China Agricultural University, Beijing, 100193 China; 2 Konge Larsen ApS, Kongens Lyngby, 2800 Denmark; 3 Institute of Animal Science and Veterinary Medicine, Shandong Academy of Agricultural Sciences, Jinan, 250100 China; 4 Animal Genomics and Improvement Laboratory, Agricultural Research Service, Henry A. Wallace Beltsville Agricultural Research Center, USDA, Beltsville, MD 20705 USA; 5 Center for Quantitative Genetics and Genomics, Aarhus University, Aarhus, 8000 Denmark

**Keywords:** Artificial insemination, Cattle, Epigenetic inheritance, Genetic improvement, Germline epigenomics

## Abstract

Sperm is essential for successful artificial insemination in dairy cattle, and its quality can be influenced by both epigenetic modification and epigenetic inheritance. The bovine germline differentiation is characterized by epigenetic reprogramming, while intergenerational and transgenerational epigenetic inheritance can influence the offspring’s development through the transmission of epigenetic features to the offspring via the germline. Therefore, the selection of bulls with superior sperm quality for the production and fertility traits requires a better understanding of the epigenetic mechanism and more accurate identifications of epigenetic biomarkers. We have comprehensively reviewed the current progress in the studies of bovine sperm epigenome in terms of both resources and biological discovery in order to provide perspectives on how to harness this valuable information for genetic improvement in the cattle breeding industry.

**Supplementary Information:**

The online version contains supplementary material available at 10.1186/s40104-023-00874-9.

## Introduction

The sperm of an elite sire can be used to breed thousands of cows to transmit genes from one generation to the next through artificial insemination (AI) in dairy cattle, where sperm epigenome influences offspring’s development, production, and fertility during transmission [1]. Thus, sperm quality is essential for successful AI. A better understanding of the epigenetic mechanism and more accurate identifications of epigenetic biomarkers are beneficial for the selection of bulls with superior sperm quality [2–6]. In practice, the results of each insemination of each bull, the mating results of each batch of frozen semen, and the phenotypes of a large number of offspring are accurately recorded. Therefore, bulls are also an excellent animal model for investigating the relationship between sperm epigenome and offspring phenotype. Here, to facilitate the utilization of sperm epigenetics in the cattle breeding industry, we provide a comprehensive review on the current progress of bovine sperm epigenome studies in terms of both resources and biological discovery focusing on four aspects: (1) epigenome of bovine sperm, (2) impacts of sperm epigenome on complex traits in cattle, (3) application of sperm epigenetic modification in cattle breeding, and (4) further potentialities and perspectives on how to harness sperm epigenetics for the genetic improvement of livestock.

## The epigenome of bovine sperm

### Bovine sperm characteristics and importance of artificial insemination in the breeding industry

#### Bovine sperm characteristics

Spermatozoon is generated through the spermatogenesis differentiation process. Contributing approximately half of the genetic information, spermatozoon merges with an ovum to form a zygote, which can finally develop into an embryo [7, 8]. In mammalian sperm cells, DNA interacts with protamines to form linear and side-by-side arrays of chromatin in a high-degree compact structure, where protamines replace the DNA-wrapped histones progressively in the histone-to-protamine transition [9, 10]. The bulls’ ejaculate volumes are similar to those of humans and sheep but less than those of pigs. Cryopreserved sperms have been used for a long time in some farm animals, but thawed sperm qualities vary among them. Bull sperm motility gradually decreases by 50% after cryopreservation, while pigs, sheep, and horse sperm show more decreased motility than bull sperm [11]. However, over the years, sperm cryopreservation techniques have improved the quality of conserved sperm [12].

#### Importance of artificial insemination in the breeding industry

AI with frozen-thawed bull semen has been implemented since the technology of semen preservation was developed [13, 14]. Currently, AI is a powerful and widely used tool for rapid genetic improvement in the dairy cattle population when superior genetics are introduced to improve the economic traits in a shorter period of time as compared to the traditional natural service. Clearly, AI allows to overcome natural barriers, utilizate superior genetics, and increase efficiency and productivity [1]. Sperm epigenomes (e.g., DNA methylation, chromatin-associated proteins and non-coding RNAs) will be partly transmitted to the embryo, leading to the so-called intergenerational and transgenerational epigenetic inheritance, to influence the early development and health of offspring [2]. Furthermore, selection, breeding, and semen processing practices for AI may potentially cause epigenetic alterations of sperms, whereas other practices like embryo technology or hormonal treatments may influence sperm epigenome in the long-term period [3]. Undoubtedly, the understanding of bovine sperm epigenome and the identification of epigenetic biomarkers of sperm quality can help the selection of superior bulls in terms of both male fertility and genetic values of other economic traits reflected in the offspring (e.g., milk production and health) [4–6].

**Fig. 1:**
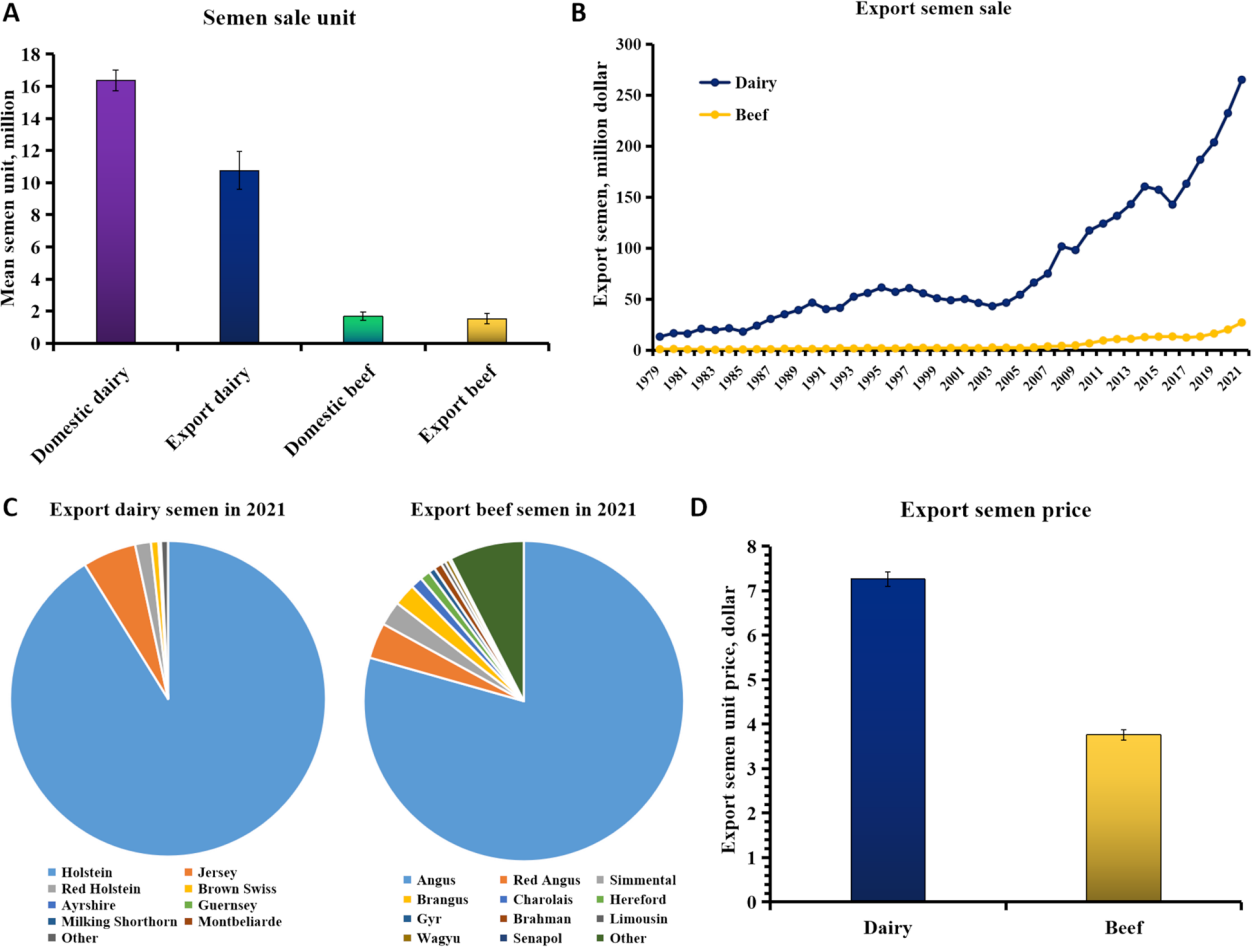
The domestic and export semen sales of dairy and beef cattle provided by the National Association of Animal Breeders (NAAB) (https://www.naab-css.org/semen-sales). **A** The sold semen unit averaged over 22 years from 1979 to 2021. Note: Bars indicate standard errors. **B** The export semen sales (dollar) of dairy and beef cattle from 1979 to 2021. **C** The percentages of export semen sales for different breeds of dairy and beef cattle in 2021. **D** The export semen price (dollar) of dairy and beef cattle averaged over 22 years from 1979 to 2021. Note: Bars indicate standard errors

For over 75 years, the National Association of Animal Breeders (NAAB) has united ~ 100 organizations from 12 countries all over the world (USA, Canada, Australia, China, Denmark, Israel, Italy, Japan, Netherlands, Spain, Switzerland, and Uruguay) to engage in the AI for promoting the mutual interests and ideals of its members. Using the datasets provided by NAAB regular members, we summarized the semen sale reports from 1979 to 2021 in Fig. 1, which includes domestic and export sales of dairy and beef cattle (https://www.naab-css.org/semen-sales). Notedly, the most productive bull produced 2.4 million semen units in its entire productive life. The summarized sold semen units for the past 22 years reached 1.3 billion, where dairy (domestic and export) takes up the most (Fig. 1A). The export of dairy semen sales increased sharply per year, and the amount reached 265 million dollars in 2021 (Fig. 1B). In 2021, a total of 30,515,959 dairy and 8,315,936 beef semen units were sold, where Holstein (91.17%) and Angus (79.38%) were the main breeds, respectively (Fig. 1C). On average, the prices of export semen units are around 7.26 dollars for dairy cattle and 3.76 dollars for beef cattle, with small fluctuations (Fig. 1D).

### Bovine sperm epigenome

**Fig. 2:**
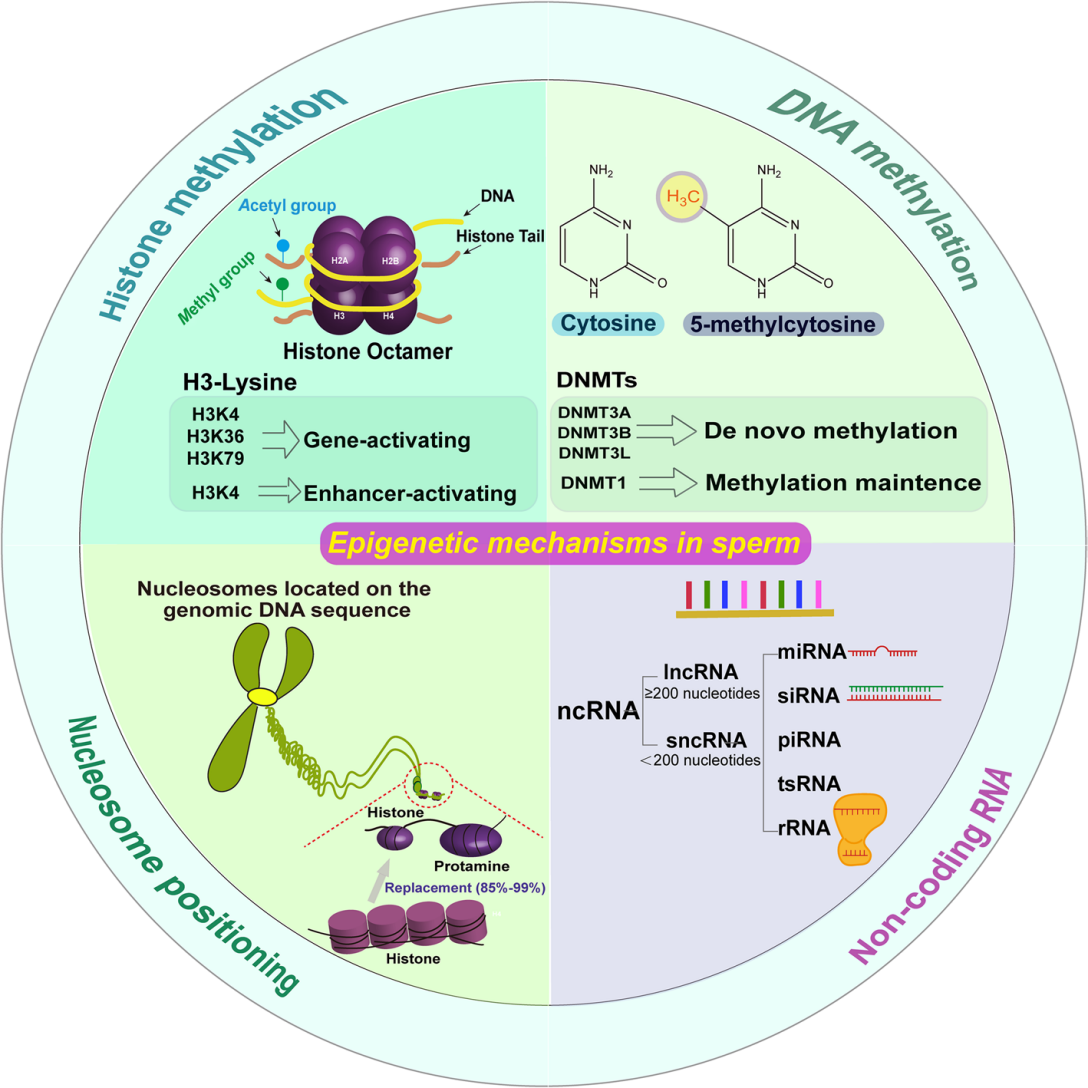
Types and mechanisms of bovine sperm epigenome. Note: ncRNA, lncRNA, sncRNA, miRNA, siRNA, piRNA, tsRNA, and rRNA represent non-coding RNA, long non-coding RNA, short non-coding RNA, microRNA, small interfering RNA, Piwi-interacting RNA, tRNA-derived small RNA, and ribosomal RNA, respectively

The differentiation process of male germ cells into functional spermatozoa is characterized by the epigenetic reprogramming via the changes of DNA methylation, chromatin (with ~ 85% to 99% histones replaced by protamines in different species), and non-coding RNAs, such as microRNAs [2, 15] (Fig. 2). The toroid-shaped structure of DNA is finally formed with arginine-rich protamines to enable a higher level of chromatin compaction [3], which helps to reduce nuclear volume and avoids oxidation during migration for fertilization of an oocyte. Therefore, spermatozoa are usually transcriptionally inactive, and their epigenome is unique as the ultimate form of male germ cell differentiation [16].

#### Epigenetic mechanisms in sperm

During male germ cell differentiation, DNA methylation is controled by the activity of the DNA methyltransferases (DNMTs), i.e., DNMT3A, DNMT3B, and their cofactor DNMT3L for the de novo methylation [17, 18], while DNA demethylases (TET enzymes) is involved in demethylation to maintain normal gene expression [19]. In addition, DNMT1 is responsible for methylation maintenance during the different phases of spermatogenesis in the adulthood period [20–22]. Histone methylation is the modification after the transfer of methyl groups to certain amino acids of histone proteins [2, 23]. The common acceptor sites for histone methylation marks are lysine and arginine residues, where histone H3 is the primary site [24]. In the general situation that does not apply to sperm, histone mono-methylation state of lysine at H3K4 is uniquely enhancer-activating, while di-methylation and tri-methylation states of lysine at H3K4, H3K36, and H3K79 are typically gene-activating, where H3K36 and H3K79 methylations primarily occur over gene bodies [25–28]. However, histone arginine methylation is more complex because of multiple nitrogen atoms and is less understood in terms of gene regulation [24]. Sperm tRNA-derived small RNAs (tsRNAs) are mainly tRNA fragments with a length of 29 to 34 nucleotides that modulate offspring phenotypes as the mediators of transgenerational inheritance for paternally acquired traits [29]. Piwi-interacting RNAs (piRNAs), the largest class of small non-coding RNA molecules [30, 31], are mostly abundant in spermatocytes, spermatids, and testicular sperm [32]. Ribosomal RNAs (rRNAs) are the primary components of ribosomes, playing crucial roles in high-quality sperms [32–34]. There are other small non-coding RNAs (sncRNAs) [e.g., small interfering RNA (siRNA), small nuclear RNA (snRNA), small nucleolar RNA (snoRNA)] in sperm, which help maintain the translational quiescent state of sperm when they are at high levels. For instance, siRNAs usually regulate expression by binding to a 3’UTR target sequence to inhibit or activate translation or target messenger RNAs (mRNAs) for degradation [33, 34]. The unique epigenetic modifications are indispensable for male germ cells’ differentiation to functional spermatozoa. So far, many studies have worked on DNA methylation in spermatozoa to compare the sperm methylation patterns of different tissues across different species [35, 36].

#### Genome-wide DNA methylation patterns of bovine sperm

In male germline cells, most DNA methylation patterns remain conserved across species. However, many highly conserved genomic regions show quite different methylation patterns that could result in the independent evolution of the genome and epigenome. One resetting of DNA methylation patterns occurs during germ cell development that finally reaches to somatic level after fertilization through the blastocyst stage. Another global resetting of DNA methylation patterns occurs during mammalian development early in embryogenesis [37]. In primates, Molaro et al. [38] suggested that the model of methylation patterns shaped genomic cytosine-guanine dinucleotide (CpG) distributions to indicate a greater influence on methylation profiles during germ cell maturation.

Comparing sperm with somatic cells in cattle, Zhou et al. showed large methylation pattern differences among common repeats, whole genomic CpGs, hypomethylated regions (HMRs), partially methylated domains (PMDs), and pericentromeric satellites, where the HMRs were observed in most sperm promoters and the high methylations in the sperm bodies of active genes, as well distinct methylation patterns around TSS [38, 39]. Fang et al. [40] found that more than 80% of genomic elements were highly methylated in the cattle and human sperm, where an obvious bimodal pattern of methylation levels was observed in promoters and CpG islands. Improper DNA methylation patterns at promoter gene regions can also favor the dysregulation of the target gene and initiate tumor transformation [41, 42]. DNA methylation patterns in male germ cells can also be altered by exposure to a deleterious environment to ultimately impair fertility [15, 36]. The altered DNA methylation patterns in bovine embryos led to hyperinsulinemia diseases, when embryos were exposed to various metabolic stresses [43].

#### Cross-species/tissue comparison reveals bovine-specific sperm DNA methylation

Cattle have a smaller effective population size and higher linkage disequilibrium (LD) among genomic variants after intensive selection over the years, but elusive genetic variations cannot fully interpret complex traits variation because they are also reflected in DNA methylation. We speculate that to some extent, DNA methylation regulations of complex traits are conserved between humans and livestock; while, genes with species-specific hypomethylated promoters are often thought to regulate species-specific traits. Recently, we performed cross-species comparisons of DNA methylome from three mammals [40]. Additionally, the relationships between DNA methylation patterns and economic characteristics have been investigated to assess the variation levels in different performances, developmental stages, and environments [44]. More future studies are required to fully explore the specificity of sperm DNA methylations that can contribute to cattle complex traits.

The conservation of high global methylation levels in sperm and the presence of cross-species hypomethylated loci suggest its important role in epigenetic modification in germ cell differentiation, sperm motility, and zygote reprogramming [45, 46]. Species-specific epigenomes improve our biological interpretations of their phenotypic diversity and adaptive evolution [47]. We reported that genes with cattle-specific hypomethylated promoters (e.g., *DGAT2*) [40] are mainly involved in lipid storage and metabolism and may influence the lineage-specific phenotypic variations, milk production, probably due to the interaction between DNA methylome and underlying nucleotide sequence or the inheritance of partial DNA methylome over generations [48]. Breed-specific HMRs of three commercial pig breeds are also reported to be related to phenotypic changes [49]. Interestingly, age-related differentially methylated regions (ageDMRs) were reported to be largely species-specific based on bisulfite pyrosequencing data from 10 regions [50]. The authors stipulated that ageDMRs in the epigenomic evolution regions may explain the lineage-specific environmental adaptations and predict the age-dependent sperm-related traits. Interestingly, genes regulated by sperm DNA methylome of differential fertility in both humans and bulls play significant roles in embryo development and aging. The role of DNA methylations of imprinted GNAS (guanine nucleotide binding protein, alpha stimulating) locus (homologous genes of mice and swine) has been reported in gametogenesis and male fertility [51]. However, additional systematic biological characteristics and conserved cross-species loci of sperms are needed to help us better understand the regulatory mechanisms of male fertility-related traits.

**Fig. 3:**
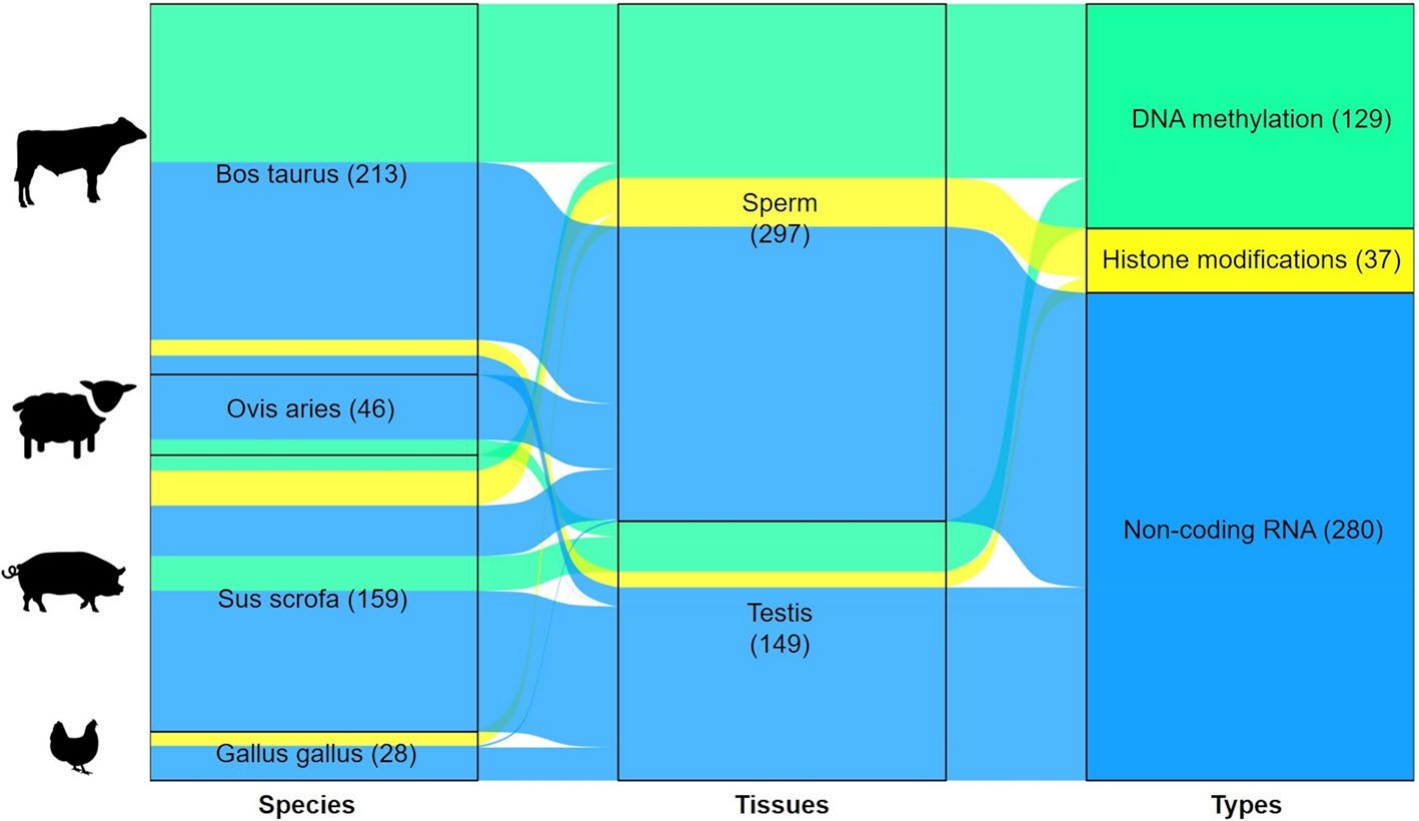
Sankey diagram of the summarized epigenomic datasets of sperm and testis tissues of livestock in Sequence Read Archive (SRA). Note: Flow describes the organization of data types and source tissues

In spite of annotation projects of regulatory elements on multiple tissues across different species, such as Roadmap Epigenomics and ENCODE for humans and FAANG and FarmGTEx for livestock, fewer epigenomic datasets of sperm samples accumulated due to their biological characteristics different from somatic tissue samples [52, 53]. From the reported epigenomic datasets of sperm and testis tissues in cattle, sheep, chicken, and pig, we found that most of the studies focused on the non-coding RNA modifications in sperms (Fig. 3) because of their easy data collection and analysis. Tissue-specific histone marks in human epigenomes can facilitate a deep understanding of epigenetic mechanisms for bovine complex traits, which is attributed to the epigenome conservation of different tissues across mammals [47]. The shared methylation quantitative trait loci (meQTLs) with different DNA methylation patterns among tissues, such as sperm and testis, may also provide an opportunity to study tissue-specific complex traits [54]. The characterization of large-scale and accurate phenotypic measurements in livestock can address the problems of ethical limitations and inconvenient direct measurements in human medical research [55].

## The impacts of sperm epigenome on complex traits in cattle

### Bovine epigenetic patterns associated with fertility and aging

Spermatogenesis is particularly vulnerable to epigenetic alterations, and aberrant sperm DNA methylation is associated with infertility [56]. During spermatogenesis, dysregulations can result in the abnormal expression of target genes to cause infertility [57, 58]. In aging males, fertility and sperm quality decrease, and DNA fragmentation rates increase [46]. In humans, sperm epigenetic alterations associated with aging can accumulate over time to potentially influence fertility [59].

#### Bovine sperm epigenetic biomarkers associated with fertility traits

In livestock, a large amount of epigenetic data has been generated, so systematic collation of epigenetic data related to sperm and annotation results of sperm epigenome can provide biological explanations for phenotypic diversities and adaptive evolution. Bovine sperm-related traits such as semen quality, fertility, etc., that are critical for bovine reproduction can be influenced by environmental, regulatory, and epigenetic factors [60]. Recent studies focus on bull fertility traits such as sire conception rate (SCR), but semen quality was rarely studied as a result of the direct discard of unqualified semen before AI [61, 62].

**Fig. 4:**
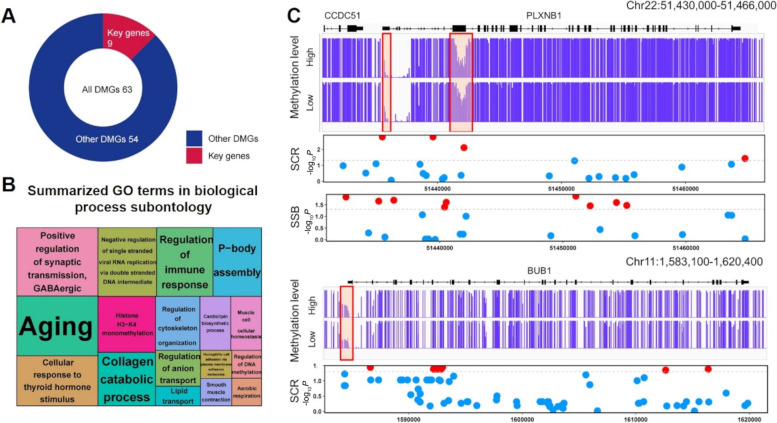
Functional annotation of DMGs associated with semen quality. **A** Nine selected key genes of 63 DMGs around GWAS signals of bovine reproduction traits. **B** Summarized GO terms of biological process for 63 DMGs. **C** Gene tracks and significant GWAS signals of bovine reproduction traits (SCR and SSB) around *PLXNB1* and *BUB1*. Note: DMGs indicate gene promoters with overlapping differentially methylated regions (DMRs). SCR and SSB indicate sire conception rate and sire stillbirth, respectively

In practice, our own study (unpublished data) collected 6 Holstein bull sperm samples for whole-genome bisulfite sequencing (WGBS) from screening DNA methylation biomarkers related to the comprehensive evaluation index of semen quality—number of motile sperm per ejaculate (NMSPE). In total, 63 genes where those promoters overlapped with NMSPE-associated differentially methylated regions (DMRs) (Additional file 1: Table S1) were significantly and selectively enriched for genome-wide association study (GWAS) signals of SCR, strength, and livability traits. Of them, nine key genes were further selected (Table 1), as they have large methylation differences and close to strong GWAS signals nearby. In the previous studies, *CATSPER4*, *DMKN*, *ELN*, *INSL3*, *LAMB2,* and *PATL2* within 20 kilobases of GWAS signals of calving fertility index (CFI) and sire calving ease (SCE) traits were reported [63–69]. Moreover, *PLXNB1* and *BUB1* were detected around significant GWAS signals of SCR trait (Fig. 4) as reported previously [70–72]. In Table 1, we also listed additional sperm epigenetic biomarkers associated with bovine/human/mouse fertility traits from other studies.

**Table 1:** Sperm epigenetic biomarkers associated with fertility from our results and other studies

Epigenetic type		Epigenetic biomarker	Tissue	Fertility trait	Reference
DNA methylation		*BUB1*, *CATSPER4*, *DMKN*, *ELN*, *INSL3*, *LAMB2*, *PATL2*, *PLXNB1*,* TMEM235*	Holstein bull sperm	Number of motile sperm per ejaculate	Our own unpublished data
		*CYP26B1*, *SNAI2*, *PLD1*, *LZTR*, *MAPK8IP3*, *NME3*, *LIG3*, *POU5F1*, *GHSR*, *SIRT1*, *LRGUK*, *RESP18*,* STX5*	Buffalo bull sperm	Cattle fertility	[73]
		*Crisp2*,* Hgf, Zfp36l1*	Holstein bull sperm	Cattle fertility (SCR)	[74]
		*SPEF2*	Holstein bull sperm	Cattle sperm motility	[75]
		*HSPA1L*, *ACTN1*, *PSMD3*,* CSRP2*	Holstein bull sperm	Sperm quality/female reproductive traits	[63]
		*EEFSEC, CYP26B1*, *SNAI2*, *PLD1*, *LZTR*, *MAPK8IP3*, *NME3*, *LIG3*, *POU5F1*, *GHSR*, *SIRT1*, *LRGUK*, *RESP18*,* STX5*	Bubalus bubalis	Cattle fertility	[76]
		*SAMD5*,* PDE5A*	Holstein bull sperm	Cattle fertility (SCR)	[46]
		*SFRP1STXBP4*, *BCR*, *PSMG4*, *ARSG*, *ATP11A*, *RXRA*	Holstein bull sperm	Cattle fertility	[62]
		*LBX1* (upstream regions), *NPAS1* (exons), *SORCS2* (introns), *PLXNB2* (intron–exon junctions), *ATG7* (3’UTRs)	Montbéliarde bulls sperm	Cattle fertility	[61]
Histone methylation		H3K4me2, H3K27me3	Buffalo	Cattle fertility	[76]
		H3K27me3, H3K27ac	Holstein bull sperm	Cattle fertility	[77]
Non-coding RNA	lncRNA	TCONS_00041733	Holstein bull sperm	Cattle sperm motility	[78]
		*COX7A2*, *COX6B2*, *TRIM37*, *PRM2*, *INHBA*, *ERBB4*, *SDHA*, *ATP6VOA2*, *FGF9*, *TCF21*	Wandong bull testes	Cattle spermatogenesis	[79]
	miRNA	miR-93, miR-106b, miR-100, miR-122, miR-184, miR-486-5p, miR-2285n	Holstein bull sperm	Cattle sperm motility	[80]
		miR-15a, miR29	Holstein bull sperm	Cattle fertility	[81]
		miR-33b, miR-126-5p, miR-205, miR-505, miR-532, miR-500, miR-542-5p, miR-216b, miR-339a	Aberdeen Angus	Cattle fertility	[82]
	siRNA	*DICER*, *DGCR8*	Mouse male sperm	Male fertility	[83]
	piRNA	piR-31068, piR-31925, piR-43771, piR-43773, piR-30198	Chinese male sperm	Male fertility	[84]
	tsRNA	tsRNA^Gln−TTGs^	Pig male sperm	Male fertility	[85]
	rRNA	*RPL23*, *RPL27A*, *RPS18*, *RPL6, RPL36AL*, *RPL37*	Human sperm	Male spermatogenesis	[86]
Nucleosome positioning		CTCF	Human sperm	Male fertility	[87]

lncRNA, miRNA, siRNA, piRNA, tsRNA, and rRNA indicate the long non-coding RNA, microRNA, small interfering RNA, Piwi-interacting RNA, tRNA-derived small RNA, and ribosomal RNA, respectively

#### Epigenetic biomarkers associated with aging traits

Aging is the process of becoming older that is characterized by the functional decline, morbidity increase, and final death. Based on DNA methylation, the epigenetic clock can be used to measure the biological age of any tissue across the entire life course and to link developmental and maintenance processes to biological aging [88]. The epigenetic clock in multiple tissues across multiple species has also been constructed to describe the relationship between global methylation levels and chronological ages and to predict aging and health in the field of precision medicine [89]. Studies of the epigenetic clock have also been conducted in a range of species (Table 2), such as humans, pigs, chickens, dogs, cats, horses, sheep, goats, deer, bats, elephants, whales, dolphins, rats, zebras, etc., to predict their ages and health statuses [90-104]. Wilkinson et al. demonstrated the accurate correlation of DNA methylation on chronological age using bat wing tissue and revealed the negative association of methylation rates at age-associated sites with longevity across different species [92]. The age and longevity-associated sites are enriched in promoter regions of genes associated with innate immunity or tumorigenesis [92]. Seale et al. summarized the linking details of DNA methylation to aging phenotypes and aimed to extend healthspan and lifespan through longevity strategies based on the alterations of DNA methylation patterns and machinery [94]. The sperm epigenetic clock is potentially utilized as a novel biomarker to predict time-to-pregnancy [105], which suggests that it can also be used as a scoring method to assess age-related traits for their true level reflection. Therefore, studying the sperm epigenetic clock and its genetic control will provide a novel and reliable biomarker for the breeding program of domestic animals, including cattle, for longevity traits [99].

**Table 2:** DNA methylation biomarkers associated with aging traits in different species

Epigenetic biomarker	Aging trait	Tissue	Species	Reference
*DENND1A, SIRT1*	Aging	Blood and oocyte	Cattle	[90]
*NKX6-1, ISL1, LHX1, MAP4K3, MCF2L* and* SMAD7*		Tail hair		[91]
Promoter regions of key transcription factors	Exceptional longevity	Wing	Bat	[92]
*PRC2*, *Hox*, *EN1*	Aging	Blood, bladder, frontal cortex, kidney, liver, and lung	Pig	[93]
*P2RXL1*, *SCGN*, *EDARADD*, *IPO8* and *NHLRC1*	Lifespan	Blood	Human	[94]
*Aqp1*, *Npy* and *Adcyap1r1*	Aging	Adipose, blood, liver, kidney, muscle, and lung	Mouse	[95]
*FANCL*, *MAF*, *ZNF608*, *PBX3*, *PLCB1*, *NEUROD1* and *BARHL2*	Aging	Blood	Zebra	[86]
*ZFHX3*, *PGM1*, *SATB2* and *KNC4*	Age	Blood	Elephant	[82]
*Igfbp3* and *DNMT3L*	Health	Jejunum, ileum, breast muscle and spleen	Chicken	[78]
*SLC12A5*, *HECTD2*, *NEUROD1* and *FOXG1*	Age	Blood	Cat	[96]
*NPAS3*, *STC1*, *HOXC4* and *LOC117200810*	Age	Blood and skin	Whale	[97]
*ANK1*, *EVX2* and *UNC5D*			Dolphin	
*FGF8*, *PAX6*, *PAX5* and *HOXC4*	Age	Blood and ear	Sheep	[98]
*TNRC6A* and *LHFPL4*	Age	Ear	Deer and goat	[99]
*LHFPL4*, *BARHL2* and *PLD5*	Age	Blood, liver and skin	Rat	[100]

### Intergenerational and transgenerational epigenetic inherited traits

In cattle, detailed pedigree records, accurate semen quality records per ejaculation, and long-term progeny testing make sperm an ideal medical model for studying intergenerational and transgenerational epigenetic inheritance related to complex traits.

#### Intergenerational and transgenerational epigenetic inheritances

Intergenerational epigenetic inheritance refers to the transmission of epigenetic alterations through the sperm or oocyte with direct exposure to next or more generations, whereas transgenerational epigenetic inheritance refers to the transmission of epigenetic alterations through the sperm or oocyte without continued direct exposure to even more generations [106–108]. True transgenerational inheritance is the transmission via sperm to the second generation when the exposure occurs in an adult individual but to the third generation if the exposure occurs in a gestating female [109]. Intergenerational inheritance is the transmission to the first generation on the paternal side or the transmission to the first and second generations when maternal environmental exposures occur [110]. The mammalian sperm epigenetic inheritance was first observed in mice for the specific alternations of *Mup* gene expressions through the paternal germ line [111]. In transgenerational epigenetic inheritance, certain effects can be epigenetically inherited in the absence of the same environmental exposures after several generations [109]. McRae et al. found that the transgenerational similarities in DNA methylation are largely caused by underlying genetic similarity with less evidence for common environmental effects [112], i.e., approximately 20% of DNA methylation differences are attributed to DNA sequence variation that is not located within CpG sites.

#### Environmental factors associated with epigenetic inheritance in sperm

Environmental factors (toxicants, abnormal nutrition, stress, etc.) can promote intergenerational and transgenerational epigenetic inheritances through epigenetic changes in sperm [113]. It has been reported that transgenerational epigenetic biomarkers of disease pathology can be used to assess disease susceptibility in sperm [114]. For example, germline epigenetic alteration due to early-life paternal exposures is anticipated to be a molecular component of autism spectrum disorder etiology [115]. At least one of the inherited chromatin signals for transcription regulation (H3K4me3, H3K27me3, CTCF, among others) is transmitted to the first mitotic cell divisions in the early embryo [116, 117]. Siklenka et al. found that severe development and survivability were impaired by *KDM1A* overexpression with a specific loss of H3K4me2 at the developmental regulatory genes, which lasted for two subsequent generations [118].

Even though sperm cryopreservation is the best way for AI after long-term preservation, it requires fertilization ability enhancement because of its negative effects on acrosomal morphology, cytoarchitecture, cell viability and survival, motility, and acrosomal enzyme activity [119, 120]. Cryopreservation can produce DNA lesions in the key epigenetic syndromes-related genes (*ADD1*, *ARNT*, *BIK*, *FSHB*, *PEG1*/*MEST*, *PRM1*, *SNORD116*/*PWSAS,* and *UBE3A*) [121], and increase histone 4 levels associated with chromatin remodeling and compaction [122]. After insemination with frozen-thawed semen, the increased cytosine methylation levels of mares lead to lower fertility rates [123]. This could be partially explained by the typical methylations of sperm induced by the cryopreservation procedure, which can be used to evaluate semen quality [36]. For example, Liu et al. observed higher sperm quality in the bull with obviously higher sperm methylation levels between monozygotic (MZ) twin AI Holstein bulls [63]. It is possible that the non-shared exposures in de novo mutations, stochasticity, and utero environment may drive the epigenetic divergences between MZ twins to influence phenotypic discordance [124, 125]. Here, we summarized the recent results about the environmental factors associated with intergenerational and transgenerational inheritances in Table 3.

**Table 3:** Environmental factors associated with intergenerational and transgenerational inheritances

Environmental factor	Environmental type	Inheritance	Generation	Epigenetic modification	Epigenetic marker	Transgenerati-onally inherited trait	Species	Reference
Stress	Chronic paternal stress	Transgenerational	F2	miRNA	miR-29c, miR-30a, miR-30c, miR-32, miR-193-5p, miR-204, miR-375, miR-532-3p, miR-698	Stress dysregulation	Mouse	[124]
	Maternal heat stress	Transgenerational	F3			Heat stress	Cow	[125]
	Poly (I:C)	Transgenerational	F2		*ARHGAP40, FGB, HRH4, PHLDA2, PODN, NTSR1* and* NMU*		Chicken	[126]
	Drought stress	Transgenerational	F6	DNA methylation	II-32B, Huhan-3	Drought resistance	Rice	[127]
Obesity	Weight loss	Intergenerational	F1	DNA methylation	*TMEM18*, *CHST8*, *SH2B1*, *BDNF*, *FTO*, *MC4R*		Human	[124]
Environmental contaminant/Endocrine disruptor	Polychlorinated biphenyls	Intergenerational	F1	DNA methylation	Alu, LINE-1, Satα		Human	[128]
	Bisphenol A	Intergenerational	F1	DNA methylation	Long interspersed nucleotide elements (LINE-1)		Human	[129]
Diet	Paternal high-fat–high-sugar Diet	Transgenerational	F2	miRNA	miR-19b	Obesity and glucose intolerance	Mouse	[130]
	High-fat diet	Transgenerational	F2	miRNA	let-7c	Body weight	Mouse	[131]
	Paternal high fat diet or low-protein diet	Transgenerational	F2	tsRNA	tsRNA^Gly(GCC)^	Metabolic disorders	Mouse	[29]
	Vitamin D deficiency	Intergenerational	F1 and F2	DNA methylation	*H19ICR*	Body weight	Mouse	[132]
	Low paternal dietary folate	Intergenerational	F1	DNA methylation	*Txndc16*, *Cav1*	Offspring abnormalities	Mouse	[133]
	Utero undernutrition	Intergenerational	F2	DNA methylation	*Lxra*	Metabolic disorder	Mouse	[134]
Diabetes	Paternal prediabetes	Intergenerational	F1 and F2	DNA methylation	Pik3ca, Pik3r1	Glucose intolerance and insulin resistance	Mouse	[135]
Freeze	Cryopreservation	Intergenerational	F0	Histone	Histone H1-DNA binding Proteins and protein-DNA disulphide bond	Alteration	Pig	[136]

F1, F2 and F3 indicate the first, second, and third filial generations from parental generation (P-generation), respectively. Note: miRNA and tsRNA indicate microRNA and tRNA-derived small RNA, respectively

**Fig. 5:**
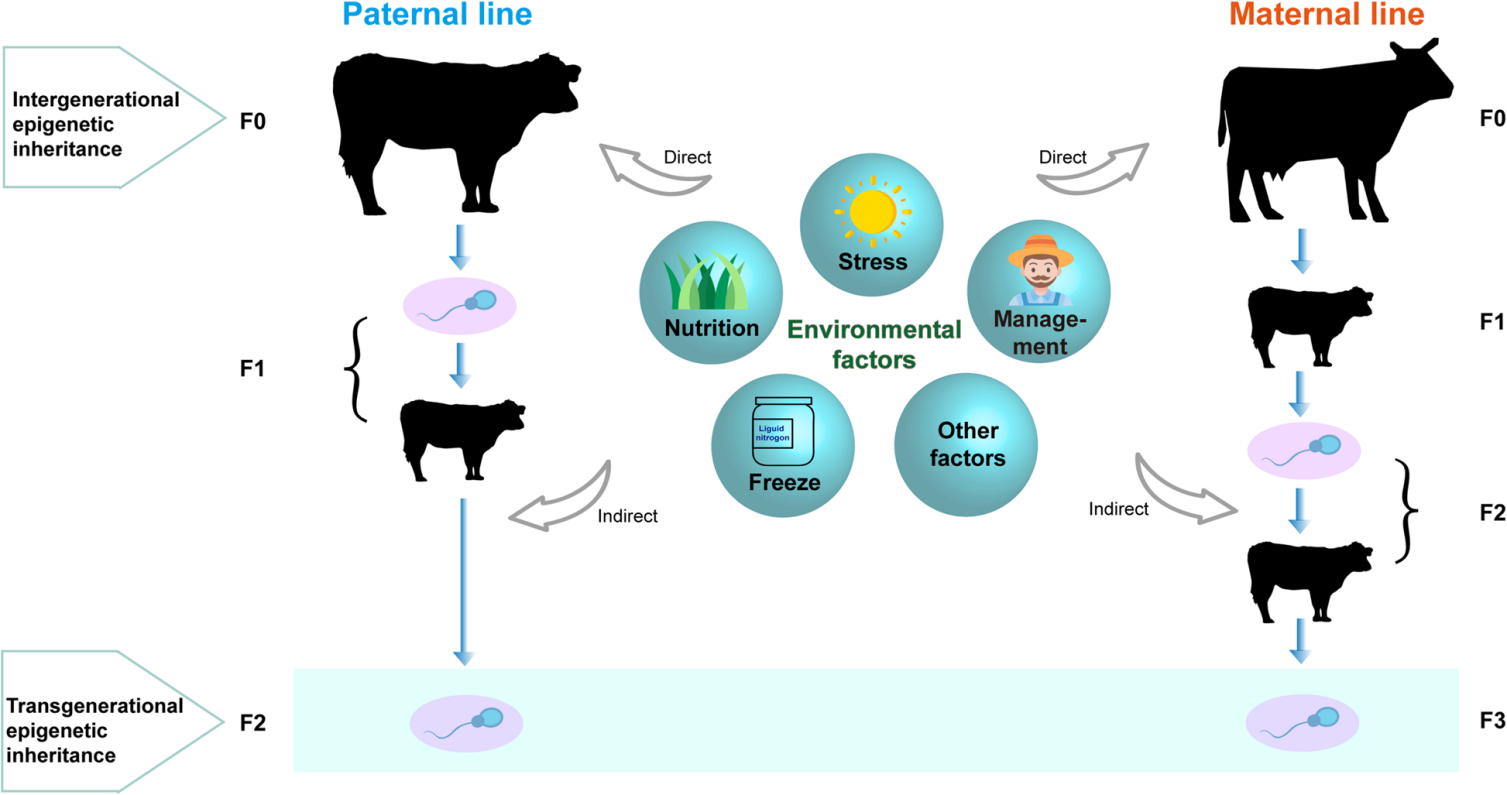
Environmental factors associated with bovine intergenerational and transgenerational inheritances

Lacal and Ventura [137] defined three types of epigenetics, i.e., a direct form of epigenetic processes (DE) and two indirect forms of epigenetic processes—within indirect epigenetics (WIE) and across indirect epigenetics (AIE). In their review, DE refers to changes that occur in the lifespan of individuals (e.g., ncRNAs mediate epigenetic processes), WIE concerns changes that occur in the womb, and AIE defines changes that happen in the predecessors before conception. Consequently, indirect epigenetic changes (WIE and AIE) are thought as intergenerational epigenetic inheritance by Lacal and Ventura [137], whereas AIE could be transgenerational epigenetic inheritance at least according to its canonical definition [109, 137, 138]. In Fig. 5, we divided the epigenetic inheritance into paternal and maternal lines, where filial generations (F0, F1, F2, and F3) were attributed to intergenerational and transgenerational inheritances with direct and indirect environmental factors such as stress, obesity, diet, freeze, diabetes, nutrition, contamination, etc. Obviously, sperm transgenerational inheritance needs at least two extra generations to be estimated (Fig. 5), so DNA methylation chip arrays would be beneficial for efficient estimations when the epigenetic markers that explain the environmental exposures are identified.

## Application of sperm epigenetic modification in cattle breeding

### Epigenetic variation associated with sperm quality for selection

#### Epigenetic variation contributing to phenotypic variation

Epigenetic variation (epi-variation) normally refers to a pure heritable variation in the absence of genetic change that corresponds with genetic variation accordingly [139, 140], where it displays relatively stable inheritance in genomic regions [141, 142]. Such heritable epi-variation could influence heritability and may potentially explain the “missing heritability” together with epistatic interactions and small-effect loci [143–146]. In some studies, the average heritability of CpGs methylation is between 5% and 19% in different tissues of humans [147–149], but some other studies reported higher heritability (19%–31%) especially for colorectum tissue [112, 150, 151]. Epigenetic changes from nearby sequence alterations are also considered as epi-variation or epimutation [152]. Garg et al. performed a survey of rare alterations in DNA methylation and obtained a catalog of rare disease-relevant epi-variations, providing insight into the underlying origins and consequences of epi-variations [153].

As sperm-related traits are complex, their heritabilities are relatively low (0.1–0.2), including the number of sperms, sperm concentration, sperm motility, and ejaculate volume [154]. Although the application of genomic selection for the bull fertility of Jersey cattle is feasible [155], the genetic improvement is probably slow due to the undefined casual epigenetic effects, for example, DNA methylations on bull fertility as shown before [60]. Theoretically, epigenetic variations could be genetically selected, but more evidence is required to identify the mutagenicity of regions subjected to environmentally-induced epigenetic variation [156, 157].

#### The rapid assessment of sperm quality with epigenetic variation

Sperm epigenetic modifications have been reported to be associated with its abnormalities. Thus, epigenetic biomarkers, especially sperm DNA methylation, could be used as an attractive quality indicator for male infertility [158]. Santi et al. identified the sperm DNA hypomethylation of *H19* and hypermethylation of *MEST* and *SRNPN* as the candidate biomarkers of male infertility [159]. Since follicle stimulating hormone (FSH) therapeutic treatment of male idiopathic infertility improved sperm numbers and motility to restore the reproductive capacity of the patient [160], Luján et al. tried to develop the molecular diagnostic approach based on the alterations in sperm DNA methylation under FSH therapy [161]. They finally identified a list of DMRs as diagnostic signatures for male infertility [162]. In practice, seminal protein-based assays of TEX101 and ECM1 have been developed for commercial clinical use, whereas ACRV1-based lateral flow immunochromatographic assay has been implemented into home tests [163–165]. Therefore, the sperm epigenetic biomarkers (Table 1) could be further developed into diagnostic arrays for bovine fertility measurement.

#### Conserved DNA methylation regions and causality of paternal experience for selection

The conserved DNA methylation regions are normally protected from being hypomethylated, where sperm promoters retain nucleosomes with hypomethylation to aid rapid activation during early embryo development after fertilization [38]. Fang et al. revealed that genes with species-conserved non-methylated promoters (e.g., *ANKS1A* and *WNT7A*) were involved in a common system and embryo development, while genes with conserved hypermethylated promoters (e.g., *TCAP* and *CD80*) were engaged in immune responses among human and cattle [40]. The conservation of tissue-specific DNA methylations across species driven by primary sequence conservation may allow comparative epigenomics to explore the biological basis of complex traits for both cattle and humans borrowing functional epigenetic annotations from each other [40, 47, 166]. These findings are consistent with other studies, showing that those epigenetic signals were largely conserved after cross-species comparison of distinct histone marks and transcriptional regulators [167]. Thus, the sperm quality related conserved epigenetic signals in unique LD with meQTLs among the different breeds would be essential to improve further cross-species selective breeding [54].

In humans, the paternal lifestyle and exposure to environmental pollution impaired semen quality causing male infertility problems. The lifestyle factors of smoking, sedentary work, alcohol, and obesity may substantially damage sperm production, where spermatogenesis is poorly organized and inefficient [168]. Kumar et al. [169] summarized that the effects of adverse environmental factors of air pollution, chemicals, and excessive heat on semen quality, including abnormal sperm morphology, decreased sperm concentration, increased sperm DNA, and reduced sperm motility fragmentation that could worsen the effects of pre-existing genetic or medical risk factors. The summarized environmental factors in Table 2 play crucial roles in bovine fertility that can be potentially used for sperm quality selection in cattle to reduce the overall incidence of infertility.

### Integrated selection of sperm quality for artificial insemination

#### DNA methylation array

**Fig. 6:**
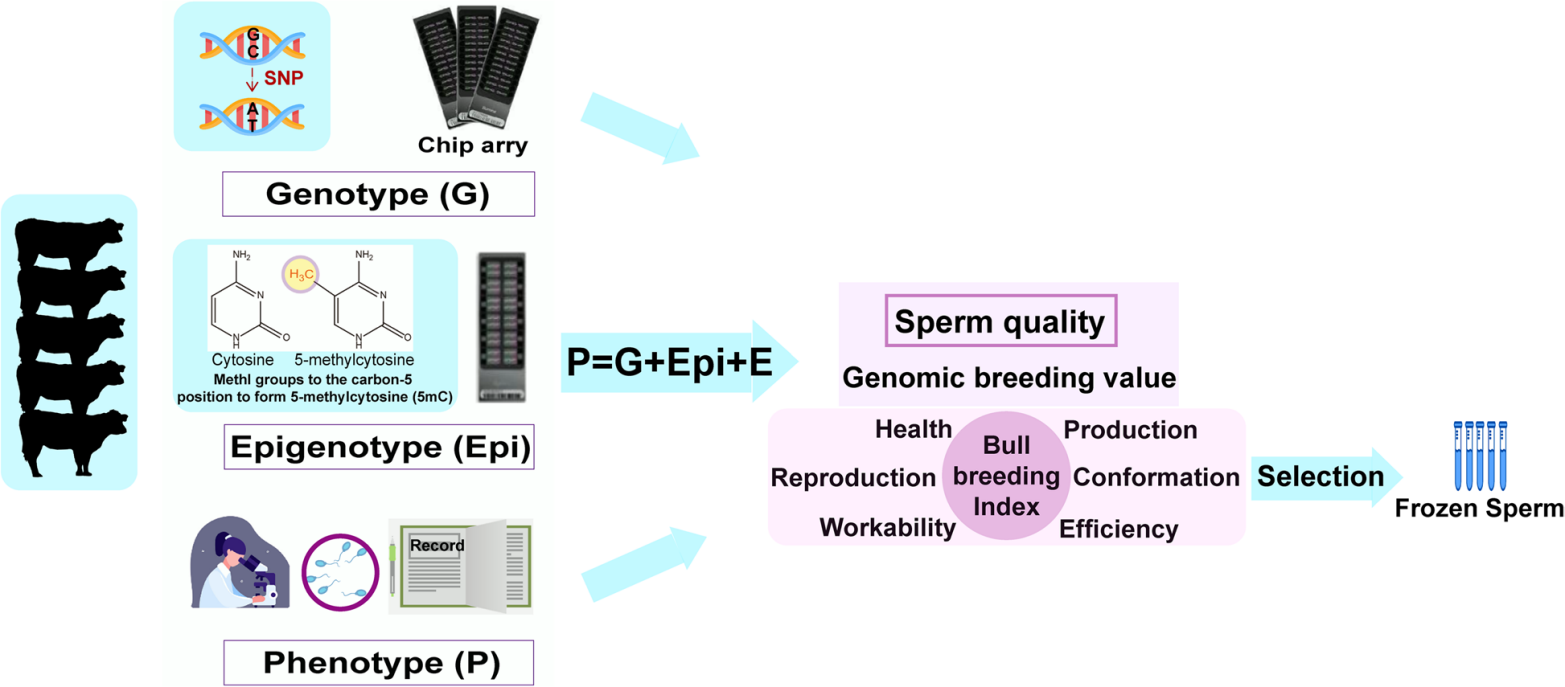
Application of epigenetic modification of bovine sperm for integrated selection of sperm quality

Several human methylation arrays have been released, such as the Illumina MethylationEPIC BeadChip microarray and Illumina Infinium HumanMethylation450 array [170]. Arneson et al. [171] recently developed a single mammalian methylation array including ~ 36k conserved CpGs that can tolerate specific cross-species mutations across over 200 species. The mammalian arrays have been used for the multi-species epigenetic clocks of epigenetic age estimations [90, 92, 93, 96–98, 100, 103, 104]. The EU Horizon 2020 project RUMIGEN (Towards improvement of ruminant breeding through genomic and epigenomic approaches) with 18 partners across EU countries, aims to develop a methylation array in order to refine genomic selection equations (https://rumigen.eu/). In cattle, O’Doherty et al. [172] used embryo compatible genome-wide epigenetics platform (only for small samples) to interrogate the global DNA methylation profiles in the different conditioned trophectoderm and embryonic discs. They found the largest impact of superovulation on the DNA methylome of subsequent embryos after the effect examinations of superovulation and in vitro system in the assisted reproduction process. Remarkably, the development of methylation arrays, including genetic and environment-derived differentially methylated sites and regions, could contribute to the new genomic selection equations for sperm quality traits (Fig. 6).

#### Integrated genomic-epigenomic selection

It is well known that the heritability of height (0.8) is higher and stabler than that of body mass index (BMI) (0.3–0.8), where BMI has more environmental contributions varying from child to adult, hence stronger epigenetic contributions than height [173–176]. Shah et al. explored the ability of DNA-methylation profiles to predict BMI and height independent of genetic contributions and found methylation profiles represent environmental effects for BMI but accounted for almost no variation in height, so they suggested combining genetic and epigenetic information for predictions of highly complex traits like BMI [177].

To fully capture the epigenetic variation that explains inheritances, environmental factors (Table 3) causing the intergenerational and especially transgenerational inheritances should also be considered during the development of epigenetic microarray (Fig. 6). In Fig. 6, epigenotype (E_epi_) representing environmental factors can be combined with genotype (G) to explain more variations of phenotype (P), so the genomic estimated breeding value (GEBV) of sperm quality is more accurate for bull selection than when only genotype (G) is used. Therefore, it is promising to use epigenetic variations to identify novel biomarkers, genes, and pathways that are not captured by the genetic variation to reflect both genetic and environmental exposures. The newly discovered CpG sites as accurate predictors have also been reported in aging-related studies [177–179]. Best linear unbiased prediction (BLUP) is widely used for phenotypic prediction. When SNP genotypes are used, it becomes genomic BLUP (GBLUP). Both BLUP and GBLUP assume all SNP effect sizes under a common Gaussian distribution. However, the assumption of a common prior distribution is perhaps unrealistic, which causes attention to relax it. Mi et al. [54] attempted to accommodate two random genetic effects, where ${G}_{epi}$ refers to random effects corresponding to genomic variants in epigenome functional elements and ${G}_{re}$ refers to random effects corresponding to the rest of the genome. Speed and Balding proposed MultiBLUP to accommodate multiple random effects, where it assigns each random effect to each region, and the genomic relationship matrix (GRM) is calculated using the SNPs in this region [180]. The different effect-size distributions may lead to the best predictions in MultiBLUP because the effect-size variances may differ in different assigned regions. Scientists could categorize the conserved epigenetic regions across species and sets of meQTLs of sperms for those regions (Fig. 6), as suggested previously [180].

Costes et al. [61] used the methylome variations to establish the predictive model using a Random Forest approach and demonstrated that the fertility status of approximately 75% of the bulls could be predicted consistently by the facultative sperm DNA methylation signature of 107 fertility-related differentially methylated cytosines (DMCs). They suggested that the less biased selected DMCs should be utilized to build the predictive model for better performance because the fertility-related DMC patterns are not conserved in all samples [61]. Based on the aforementioned methylation arrays, the new genetic merit estimations for sperm quality could be obtained by considering SNPs and DMCs simultaneously in the refined genomic selection equations (Fig. 6), where random epigenetic effects that explain the unmeasured environmental exposures complement the random genetic effects captured by the SNP arrays. Therefore, to meet the final breeding objectives, the weights of sperm quality need to be balanced with other economic traits (health, reproduction, production, efficiency, conformation, and workability) in the breeding index for the overall genetic merit score (Fig. 6).

## Further potentialities and perspectives

### Omics in bovine sperm

Recent findings of omics studies provided candidate fertility biomarkers to predict the fertility potential of young bulls for AI programs [181]. Such identified biomarkers could be used to exclude subfertile bulls that may pass the traits to future generations. For example, genomics studies found SNP variants in *MAP1B* associated with a high conception rate and SNP variants in *FSHβ* associated with a low conception rate and semen quality [182, 183]. The over-representation of PEBP4 (phosphatidylethanolamine-binding protein 4) was found in the sperms of high fertility bulls by proteomics studies [184]. Phospholipase A2 and spermadhesin also explained a significant proportion of the variations in fertility scores of dairy bulls [185, 186]. The negative correlations of seminal plasma proteins clusterin and ubiquitin with bull fertility were utilized as useful markers for poor-quality ejaculates [186]. Metabolomics studies found both low levels of citrate and isoleucine and high levels of tryptamine, taurine, and leucine in the seminal plasma of high-fertility bulls [187]. Menezes et al. [188] demonstrated that the abundances of benzoic acid, carbamate, gamma-aminobutyric acid, lactic acid, and palmitic acid were statistically different between fertility groups using bovine sperm metabolome data. Promisingly, the integrated omics analysis could contribute to identifying more multiple-layer biomarkers, but the integration statistics under the appropriate hypothesis are challenged.

### Detailed molecular phenotyping and QTL mapping in sperm

The epigenome is dynamic and tissue-specific, and the epigenetic profiles of the germ cells change during the different stages of spermatogenesis [189]. Five windows of susceptibility were identified to alter epigenetic modifications in the development of the paternal germline cells: paternal embryonic development, paternal prepuberty, spermatogenesis, periconception and post-testicular sperm maturation, and paternal development [190, 191]. Single-cell sequencing can further investigate the key genes of spermatogenesis at the individual cell resolution profile. Of note, thousands of candidate CNVs have been identified from single sperm genomes from two Holstein bulls [192]. DNA methylation could also be referred to as the “phenotype” of the gene at the level of the structure and function, so the longitudinal machine learning (ML) method can be used for dynamic repeated epigenetic profiling in different stages to predict the posterior probabilities. In order to infer the pathway activities, pre-selected reporter genes in the signaling pathways can be quantified to characterize the modulations of pathway activities induced by perturbations [193–195]. The impacts of epigenomics on molecular phenotyping will be needed to be explored when the chip array for different types of epigenetic modification (e.g., DNA methylation) becomes available and are applied in large populations.

### Epigenome editing flips genetic on–off switches

Epigenome editing aims to epigenetically modify the specific sites to turn on/off the gene expressions, which is considered as a potentially safer and more flexible way than gene editing that changes the actual DNA sequence. Kungulovski and Jeltsch reviewed the epigenome editing of chromatin modification at specific genomic loci [196]. They showed that it is necessary to find out the most promising chromatin modifications, revealing the dynamic effects of chromatin marks [196]. As far as DNA methylation is concerned, Liu et al. [197] showed the capability of dCas9-Tet1 and -Dnmt3a of precise methylation editing in mice, while Huang et al. [198] used the dCas9-SunTag-DNMT3A to amplify the concentrations of local DNMT3A that can dramatically increase the CpG methylations at the *HOXA5* locus. Gjaltema and Rots reviewed the applications of epigenetic editing to DNA methylations and histones in mammals [199]. With the CRISPR/Cas9 revolution, CRISPR-based epigenomic editing tools enable probing epigenetic alterations in both a site-specific and high-throughput manner [200]. However, it’s still a long way that epigenome editing becomes a precise tool for future applications. The application of epigenome editing to the male germline could be realized in DNA methylations [197–200] but histones, as sperm histones are largely replaced by protamines.

## Conclusions

The quality of bovine sperm is essential for successful AI worldwide. More accurate identifications of epigenetic biomarkers and integrated genomic-epigenomic selection with epigenetic chip arrays using the new genomic selection equation are required to facilitate the selection of bulls with superior sperm quality based on a better characterization of bovine sperm epigenome. Combining genetic information and other multiple omics with epigenomics is a promising way to potentially improve selective breeding using superior bovine sperm. In this review, we summarized the epigenetic biomarkers associated with fertility and aging traits and the environmental factors influencing epigenetic patterns to derive useful application information for sperm quality detection and selection. To systematically integrate existing DNA methylation markers with economic traits, new biotechnologies such as epigenetic chip arrays and epigenome-wide editing are warranted. Especially, the integrated genomic-epigenomic selection by considering SNPs and DMCs simultaneously based on the developed DNA methylation arrays in the new genomic selection equations could result in new genetic merit estimations for sperm quality, where the weights need to be redefined with other economic trait weights in the breeding index that aims to meet the overall breeding objectives.

## Supplementary Information

**Additional file 1: Table S1.** Summary of 63 DMGs (*P*-value < 0.05).

## Data Availability

Not applicable.

## Abbreviations

ageDMR: Age-related differentially methylated region
AI: Artificial insemination
AIE: Across indirect epigenetics
BLUP: Best linear unbiased prediction
BMI: Body mass index
CFI: Calving fertility index
CpG: Cytosine-guanine dinucleotide
DE: Direct epigenetic process
DMC: Differentially methylated cytosine
DMR: Differentially methylated region
DNMT: DNA methyltransferase
epi-variation: Epigenetic variation
FSH: Follicle stimulating hormone
GBLUP: Genomic BLUP
GEBV: Genomic estimated breeding value
GNAS: Guanine nucleotide binding protein, alpha stimulating
GRM: Genomic relationship matrix
GWAS: Genome-wide association study
HMR: Hypomethylated region
LD: Linkage disequilibrium
meQTL: Methylation QTL
ML: Machine learning
mRNA: Messenger RNA
MZ: Monozygotic
NAAB: National Association of Animal Breeders
NMSPE: Number of motile sperm per ejaculate
PEBP4: Phosphatidylethanolamine-binding protein 4
PMD: Partially methylated domain
piRNA: Piwi-interacting RNA
rRNA: Ribosomal RNA
SCE: Sire calving ease
SCR: Sire conception rate
snRNA: Small nuclear RNA
sncRNA: Small non-coding RNA
snoRNA: Small nucleolar RNA
siRNA: Small interfering RNA
tsRNA: TRNA-derived small RNA
WGBS: Whole-genome bisulfite sequencing
WIE: Within indirect epigenetics
