# Correction: Harnessing male germline epigenomics for the genetic improvement in cattle

**DOI:** 10.1186/s40104-023-00917-1

**Published:** 2023-08-02

**Authors:** Xiao Wang, Wenlong Li, Xia Feng, Jianbin Li, George E. Liu, Lingzhao Fang, Ying Yu

**Affiliations:** 1 Laboratory of Animal Genetics and Breeding, Ministry of Agriculture and Rural Affairs of China, National Engineering Laboratory of Animal Breeding, College of Animal Science and Technology, China Agricultural University, Beijing, 100193 China; 2 Konge Larsen ApS, 2800 Kongens Lyngby, Denmark; 3 Institute of Animal Science and Veterinary Medicine, Shandong Academy of Agricultural Sciences, Jinan, 250100 China; 4 Animal Genomics and Improvement Laboratory, Agricultural Research Service, Henry A. Wallace Beltsville Agricultural Research Center, USDA, Beltsville, MD 20705 USA; 5 Center for Quantitative Genetics and Genomics, Aarhus University, 8000 Aarhus, Denmark

**Correction: J Anim Sci Biotechnol 14, 76 (2023)**

**https://doi.org/10.1186/s40104-023-00874-9**

Following publication of the original article [1], the authors reported a typo in the author’s name and a typo in Fig. 1.

The author’s name “Jianbin Li” was mistakenly typed as “Jianbing Li” and has been corrected.

The word “demen” in Fig. 1C should be changed to “semen”.

The correct Fig. 1 should read:


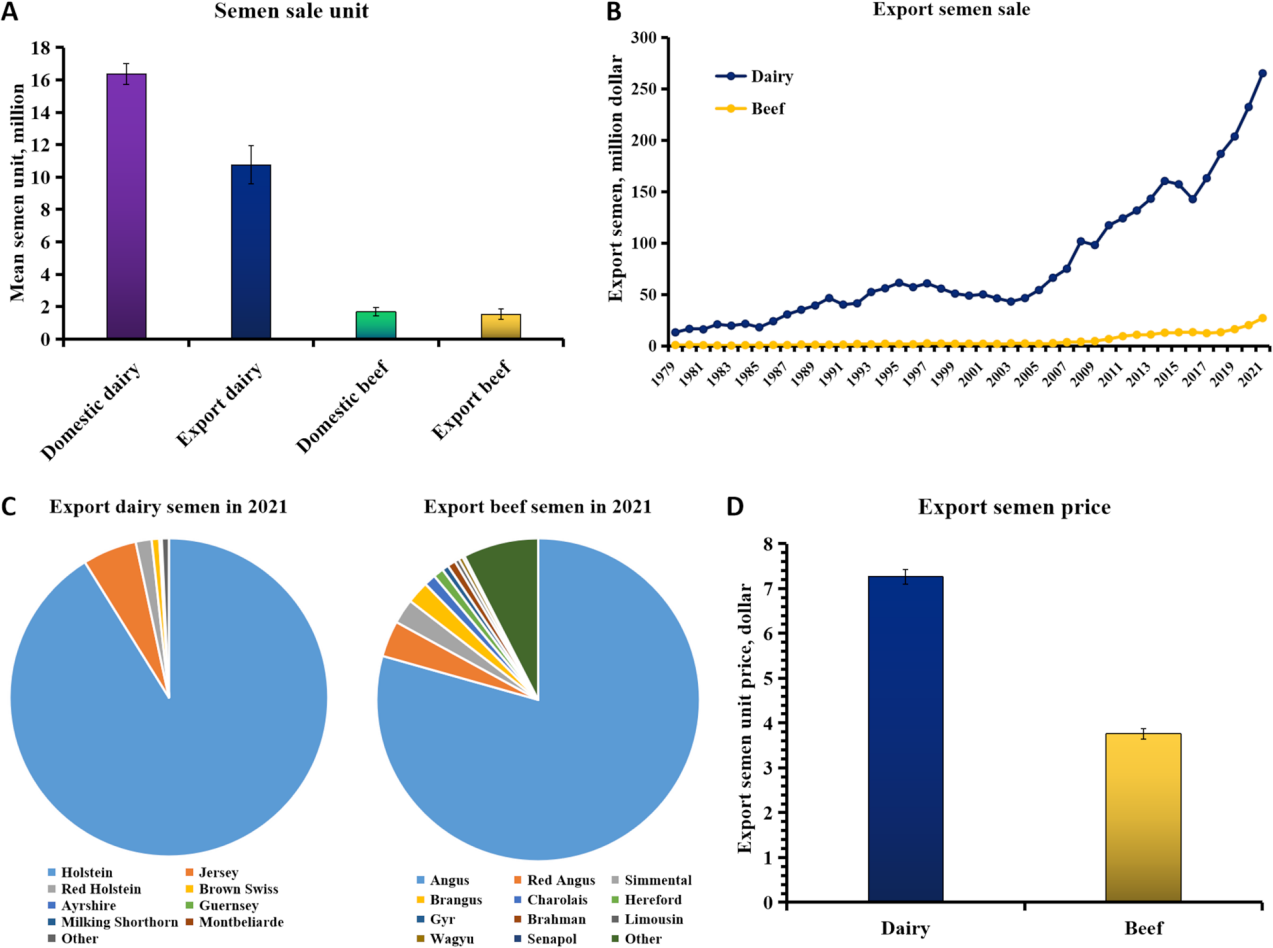


The original article [1] has been updated.
