# Supplementary material for: Harnessing male germline epigenomics for the genetic improvement in cattle
Source: J Anim Sci Biotechnol. 2023 Jun 6;14:76. doi: 10.1186/s40104-023-00874-9 (PMC10242889; doi:10.1186/s40104-023-00874-9)
Supplement: Supplementary file 1 — Additional file 1: Table S1. Summary of 63 DMGs (P-value < 0.05). [file 40104_2023_874_MOESM1_ESM.docx]

**Table S1** Summary of 63 DMGs (*P*-value < 0.05)

| **Chromosome** | **Start** | **End** | **Gene name** | **Start of DMR** | **End of DMR** | **Methylation difference** |
| --- | --- | --- | --- | --- | --- | --- |
| 1 | 66091529 | 66095529 | *ENSBTAG00000052248* | 66092639 | 66092816 | -0.26 |
| 1 | 83417537 | 83421537 | *ENSBTAG00000014449* | 83419971 | 83420170 | -0.13 |
| 1 | 104349156 | 104353156 | *ENSBTAG00000027213* | 104350995 | 104351421 | 0.31 |
| 1 | 153541136 | 153545136 | *ENSBTAG00000051752* | 153542147 | 153542483 | -0.16 |
| 2 | 6276630 | 6280630 | *MSTN* | 6279304 | 6279435 | -0.25 |
| 2 | 90334390 | 90338390 | *CDK15* | 90336290 | 90336509 | -0.28 |
| 2 | 121573674 | 121577674 | *ensembl* | 121573465 | 121573882 | -0.05 |
| 2 | 121573160 | 121577160 | *bta-mir-2887-2* | 121573465 | 121573882 | -0.05 |
| 2 | 126908286 | 126912286 | *CATSPER4* | 126911402 | 126912810 | 0.33 |
| 2 | 136021505 | 136025505 | *U6* | 136025457 | 136025677 | -0.33 |
| 3 | 21046759 | 21050759 | *U1* | 21047666 | 21047877 | -0.27 |
| 3 | 30282222 | 30286222 | *ENSBTAG00000048807* | 30284512 | 30285123 | -0.32 |
| 5 | 101222468 | 101226468 | *AICDA* | 101223136 | 101223666 | -0.25 |
| 4 | 32562174 | 32566174 | *RUNDC3B* | 32566129 | 32566390 | -0.26 |
| 4 | 52544378 | 52548378 | *ENSBTAG00000052168* | 52546057 | 52546366 | -0.21 |
| 4 | 117161402 | 117165402 | *ENSBTAG00000051995* | 117162744 | 117162784 | -0.54 |
| 8 | 83556696 | 83560696 | *CTSV* | 83560092 | 83560398 | -0.28 |
| 7 | 5323748 | 5327748 | *INSL3* | 5325728 | 5326122 | -0.31 |
| 11 | 1583412 | 1587412 | *BUB1* | 1584826 | 1585066 | -0.20 |
| 11 | 24200422 | 24204422 | *bta-mir-12030* | 24202307 | 24202478 | -0.21 |
| 11 | 94832255 | 94836255 | *ENSBTAG00000048688* | 94834091 | 94834422 | -0.23 |
| 9 | 4619918 | 4623918 | *ENSBTAG00000054972* | 4622744 | 4622861 | -0.20 |
| 9 | 15720259 | 15724259 | *IMPG1* | 15723273 | 15723481 | -0.25 |
| 9 | 57573431 | 57577431 | *ENSBTAG00000055091* | 57575439 | 57575925 | 0.26 |
| 10 | 103063307 | 103067307 | *PATL2* | 103063481 | 103063827 | -0.35 |
| 12 | 36056870 | 36060870 | *ENSBTAG00000051110* | 36058467 | 36058762 | -0.24 |
| 12 | 82557911 | 82561911 | *ENSBTAG00000052117* | 82559469 | 82559580 | -0.14 |
| 12 | 86679453 | 86683453 | *DCUN1D2* | 86679622 | 86679890 | 0.27 |
| 15 | 49291503 | 49295503 | *ENSBTAG00000054208* | 49291237 | 49291777 | -0.28 |
| 15 | 73646160 | 73650160 | *ENSBTAG00000048487* | 73647969 | 73648320 | -0.26 |
| 15 | 73646217 | 73650217 | *bta-mir-670* | 73647969 | 73648320 | -0.26 |
| 14 | 56946115 | 56950115 | *ENSBTAG00000008257* | 56948127 | 56948454 | 0.22 |
| 14 | 77010297 | 77014297 | *CA2* | 77010625 | 77011077 | 0.21 |
| 16 | 64695393 | 64699393 | *APOBEC4* | 64696682 | 64696893 | -0.24 |
| 17 | 17472357 | 17476357 | *ENSBTAG00000055020* | 17475545 | 17475747 | -0.15 |
| 17 | 17834577 | 17838577 | *MAML3* | 17838077 | 17838272 | -0.27 |
| 17 | 66242471 | 66246471 | *TPST2* | 66243213 | 66243439 | 0.15 |
| 20 | 20012934 | 20016934 | *ENSBTAG00000055189* | 20014358 | 20014607 | -0.25 |
| 20 | 20012955 | 20016955 | *PDE4D* | 20014358 | 20014607 | -0.25 |
| 21 | 14209683 | 14213683 | *ENSBTAG00000022990* | 14211178 | 14211433 | -0.38 |
| 21 | 19648107 | 19652107 | *ENSBTAG00000050172* | 19649623 | 19649921 | -0.21 |
| 21 | 44979500 | 44983500 | *U1* | 44979585 | 44980316 | 0.23 |
| 18 | 46176470 | 46180470 | *DMKN* | 46176310 | 46176601 | -0.36 |
| 18 | 56742210 | 56746210 | *C18H19orf81* | 56744278 | 56744553 | -0.25 |
| 18 | 57092648 | 57096648 | *ensembl* | 57093889 | 57093941 | -0.26 |
| 18 | 57467952 | 57471952 | *SIGLEC10* | 57468541 | 57469028 | -0.30 |
| 18 | 60654231 | 60658231 | *ENSBTAG00000011441* | 60656092 | 60656326 | -0.22 |
| 19 | 53931612 | 53935612 | *TMEM235* | 53933339 | 53933502 | -0.42 |
| 19 | 61491353 | 61495353 | *ABCA9* | 61492928 | 61493277 | -0.28 |
| 22 | 9638621 | 9642621 | *ARPP21* | 9640470 | 9640694 | -0.25 |
| 22 | 25262487 | 25266487 | *CNTN6* | 25263625 | 25263895 | -0.29 |
| 22 | 50881645 | 50885645 | *LAMB2* | 50882917 | 50883174 | -0.31 |
| 22 | 51437234 | 51441234 | *PLXNB1* | 51440993 | 51441216 | 0.32 |
| 22 | 55845377 | 55849377 | *TAMM41* | 55848879 | 55849092 | 0.23 |
| 23 | 29441488 | 29445488 | *ENSBTAG00000039534* | 29442991 | 29443188 | -0.23 |
| 23 | 30566915 | 30570915 | *ZSCAN16* | 30567472 | 30567722 | -0.25 |
| 29 | 2633494 | 2637494 | *FAT3* | 2634635 | 2634927 | 0.21 |
| 29 | 2653463 | 2657463 | *ENSBTAG00000049527* | 2653375 | 2653634 | -0.24 |
| 29 | 35917970 | 35921970 | *U6* | 35918644 | 35918919 | -0.35 |
| 29 | 41175115 | 41179115 | *ENSBTAG00000050000* | 41174851 | 41175205 | -0.30 |
| 29 | 43553590 | 43557590 | *TIGD3* | 43555776 | 43556035 | -0.27 |
| 25 | 27479274 | 27483274 | *COX6A2* | 27479843 | 27480209 | -0.27 |
| 25 | 33308763 | 33312763 | *ELN* | 33310245 | 33310500 | -0.37 |

Note: DMGs indicate gene promoters with overlapping differentially methylated regions (DMRs). The reference genome used here is *Bos taurus* (ARS-UCD1.2/bosTau9)
